# Phase Inversion-Based Doxycycline Hyclate-Incorporated Borneol In Situ Gel for Periodontitis Treatment

**DOI:** 10.3390/gels9070557

**Published:** 2023-07-07

**Authors:** Nutdanai Lertsuphotvanit, Sarun Tuntarawongsa, Takron Chantadee, Thawatchai Phaechamud

**Affiliations:** 1 Program of Pharmaceutical Technology, Faculty of Pharmacy, Silpakorn University, Nakorn Pathom 73000, Thailand; lertsuphotvanit_n@silpakorn.edu; 2 Pharmaceutical Intellectual Center “Prachote Plengwittaya”, Faculty of Pharmacy, Silpakorn University, Nakhon Pathom 73000, Thailand; 3 Natural Bioactive and Material for Health Promotion and Drug Delivery System Group (NBM), Faculty of Pharmacy, Silpakorn University, Nakhon Pathom 73000, Thailand; takron.chantadee@cmu.ac.th; 4 Department of Pharmaceutical Sciences, Faculty of Pharmacy, Chiang Mai University, Chiang Mai 50200, Thailand; 5 Department of Industrial Pharmacy, Faculty of Pharmacy, Silpakorn University, Nakhon Pathom 73000, Thailand

**Keywords:** borneol, triacetin, in situ forming gel, periodontitis, doxycycline hyclate

## Abstract

Borneol has been successfully employed as a gelling agent for in situ forming gel (ISG). While 40% borneol can regulate drug release, there is interest in novel approaches to achieve extended drug release, particularly through the incorporation of hydrophobic substances. Herein, triacetin was selected as a hydrophobic additive solvent for doxycycline hyclate (Dox)-loaded 40% borneol-based ISGs in *N*-methyl-2-pyrrolidone (NMP) or dimethyl sulfoxide (DMSO), which were subsequently evaluated in terms of their physicochemical properties, gel formation morphology, water sensitivity, drug release, and antimicrobial activities. ISG density and viscosity gradually decreased with the triacetin proportion to a viscosity of <12 cPs and slightly influenced the surface tension (33.14–44.33 mN/m). The low expelled force values (1.59–2.39 N) indicated the convenience of injection. All of the prepared ISGs exhibited favorable wettability and plastic deformation. Higher gel firmness from ISG prepared using NMP as a solvent contributed to the ability of more efficient controlled drug release. High triacetin (25%)-loaded ISG retarded solvent diffusion and gel formation, but diminished gel firmness and water sensitivity. ISG containing 5% triacetin efficiently prolonged Dox release up to 10 days with Fickian diffusion and presented effective antimicrobial activities against periodontitis pathogens such as *Porphyromonas gingivalis* and *Aggregatibacter actinomycetemcomitans*. Therefore, the Dox-loaded 40% borneol-based ISG with 5% triacetin is a potential effective local ISG for periodontitis treatment.

## 1. Introduction

Periodontitis, a significant oral health disease affecting a considerable portion of the global population (20–50%) [1], has witnessed a rise in prevalence, with severe cases now comprising up to 11% worldwide [2]. The etiology of periodontitis can be attributed to imbalanced microbial biofilms, dysregulated host immune responses, and environmental factors, including poor oral hygiene and tobacco use, leading to infection and inflammation of the gingival tissues and alveolar bone [2,3]. Clinical manifestations commonly associated with periodontitis include discomfort, bleeding of the gums, swelling of the gums, and gradual deterioration of the supporting structures of the teeth, leading to bone loss, receding gums, heightened tooth mobility, and, eventually, tooth loss [2]. Among the pathogens implicated in this disease, Gram-negative bacteria, particularly anaerobic species such as *Aggregatibacter actinomycetemcomitans, Porphyromonas gingivalis,* and *Treponema denticola,* are prevalent [4]. Furthermore, other microorganisms, including enterobacteria (e.g., *Escherichia coli, Pseudomonas aeruginosa*, and *Klebsiella pneumoniae*), *Candida albicans,* and *Filifactor alocis* have been associated with inflammation and tissue destruction [5,6]. Additionally, *Staphylococcus aureus* has been detected within periodontal pockets [7]. Hence, effective periodontal treatment approaches involving mechanical scaling, root planing, and adjunctive medication are essential for the elimination or suppression of bacterial growth on the tooth surface and within the periodontal pocket [8,9].

In the treatment of periodontal disease, antimicrobial agents are frequently employed for the elimination of infection [10]. The medication therapy for patients with periodontitis is divided into systemic and localized antimicrobial drug delivery systems. Currently, many localized antimicrobial drugs have been studied for periodontal treatment in order to minimize antibiotic resistance and the harmful side effects caused from oral administration [11]. Doxycycline hyclate (Dox) is a distinguished antimicrobial drug for the localized treatment of periodontitis and is a broad-spectrum antibiotic in the tetracycline group against Gram-negative and Gram-positive bacteria, including beta-lactamase-producing strains that occur in deep periodontal pockets [12]. A small dose of Dox taken orally can significantly and beneficially decrease gum inflammation. However, Dox has several side effects, including nausea, diarrhea, and rash from oral administration into systemic circulation [13]. To overcome these disadvantages, localized Dox delivery has been applied to provide a high dose of antimicrobial drugs at the target site. Additionally, the treatment of periodontitis using a local Dox-loaded delivery system for 4 months can significantly reduce the depth of the periodontal pocket, which is equally as effective as scaling or root planning [14]. Atridox^®^, a well-known product, contains Dox as an active compound dissolved in a polymer solution of an injectable dosage form to prolong drug release [15]. This formulation comprises a gel-forming agent dissolved in an organic solvent and transforms from a liquid dosage form to a gel-like matrix. This dosage form is currently prepared as an in situ forming gel (ISG) or in situ forming matrix. When the prepared formulation contacts crevicular fluid in the crevicular pocket, the organic solvent from the formulation diffuses into the environment, while the crevicular fluid diffuses into the formulation and induces gel formation because of the water-insoluble properties of the gel-forming agent [16,17,18]. The drug molecules in the gel matrix are gradually released over a period of time. This controlled release is achieved through a process called solvent exchange, which triggers a phase inversion and results in the formation of a gel [19]. Atridox^®^ can prolong drug release for 7 days [20]. This commercial product uses PLGA as a polymer. Therefore, in our formulation, borneol was selected as the gelling or matrix forming agent, as it is inexpensive and has an apparent low aqueous solubility as well as being biocompatible, biodegradable, and safe for long-term use [18].

Borneol (C_10_H_18_O, molecular weight = 154.25) is a highly lipid-soluble (log K_o/w_ = 2.85) monoterpenoid compound. Although it has water-insoluble properties, borneol can be soluble in various organic solvents [21,22]. The US Food and Drug Administration (FDA) has approved borneol as a flavoring agent because of its nontoxicity [23]. Importantly, borneol has antibacterial, antinociceptive, and anti-inflammatory activities [24] and can decrease the blood–brain barrier intercellular tight junctions and enhance blood-brain barrier permeability [25], as well as enhance the in vivo oral absorption of ginsenosides [26]. Moreover, borneol exhibits an antifibrotic activity in the oral cavity and has been proven safe as a penetration enhancer for the treatment of oral submucous fibrosis [27]. The low skin irritation of borneol has been reported in human volunteers and mice [28]. Borneol can be used safely for long-term use and has a low aqueous solubility, but it is miscible in some organic solvents and thus can serve as a gelling of ISG for periodontitis treatment. A previous study [18] showed that ISGs formulated with low concentrations of borneol could not create a dense gel, whereas ISG with a borneol concentration of >40% *w/w* showed a rapid gel formation that transformed into gel before filling into the crevicular pocket, and may not subsequently fit this area. Consequently, 40% *w/w* borneol represented the appropriate concentration to employ as a gel formation for ISG. Similarly, borneol-based ISG has demonstrated a sustained drug release as well as a potent antimicrobial activity against a variety of microorganisms. [18]. Even though 40% borneol can modulate drug release, novel approaches to prolong drug release are being investigated. One possible strategy is to incorporate hydrophobic vehicles (e.g., ethyl benzoate, triacetin, and benzyl benzoate) into the formulation system [29,30]. In this particular study, triacetin was chosen as an additional solvent to slow down the process of solvent exchange and to extend the release of the drug.

Triacetin (C_9_H_14_O_6_, molecular weight = 218.2) is a nontoxic edible fatty acid that can be used as an additive solvent to retard drug release [31,32]. Triacetin is moderately soluble in water (around 7%) but it is soluble in relatively hydrophobic organic solvents (log K_o/w_ = 0.25) [31,33]. Triacetin has been used as a hydrophobic solvent for ISG preparation for promoting a slow-forming phase inversion [29,33]. Solvents that possess a water solubility of <7% can produce a slower drug release through reduced water uptake [34]. In the ISG system, the addition of triacetin as a cosolvent to a polymer-based hydrophilic solution could reduce the solvent affinity between water and polymer, slowing the rate of phase inversion and a significantly reducing the overall release rate and not just the burst release [29]. Recently, Zingermann and Chern reported a combination of glycerol formal and triacetin as a solvent to prolong the blood level of fipronil (flea adulticide) for 12 months after subcutaneous injection [35]. Given these characteristics, triacetin was employed as an additional solvent in Dox-loaded borneol-based ISG formulation in this study.

This study presents the development of phase inversion-based Dox-incorporated borneol ISGs. Two solvents, namely *N*-methyl-2-pyrrolidone (NMP) and dimethyl sulfoxide (DMSO), were employed during the formulation process, while triacetin was chosen as an additive solvent. The primary objective of this investigation was to examine the impact of triacetin on the performance of borneol-based ISGs. The prepared formulations underwent a comprehensive assessment of their physicochemical properties, including parameters such as appearance, density, viscosity, wettability, surface tension, injectability, and mechanical properties. Moreover, the gel formation and microscopic changes were characterized, along with the sensitivity to water-induced gel formation. Additionally, the drug release and antimicrobial activity were evaluated to further comprehend the behavior of the formulations and a suitable concentration of triacetin within the ISG formulation.

## 2. Results and Discussion

### 2.1. Appearance, Density, Viscosity, and Surface Tension

All of the borneol-based ISG formulations and control solutions appeared as homogeneous transparent solutions. There was no precipitation or cloudy characteristic in the prepared preparations, signifying the compatibility of the components in the formulations. The physicochemical properties of each formulation, including density, viscosity, and surface tension, are shown in Table 1. The densities of triacetin, NMP, and DMSO were 1.1472, 1.0283, and 1.0924 g/cm^3^, respectively. The densities of Dox-loaded formulations (N1 or D1) were higher than those of pure solvent (NMP or DMSO) or borneol-based ISGs because of the low density of borneol (1.01 g/cm^3^) [18]. Based on the NMP series, the density of ISG gradually decreased with the proportion of triacetin, as shown for the N3 to N8 formulations, because of the hydrophobic and oily liquid properties of triacetin [32]. Notably, the density of N4 and N8 was significantly higher (*p* < 0.05) than that of N2. Similarly, the ISG formulations employing DMSO as a solvent presented a significantly lower density (*p* < 0.05) when adding more triacetin. All of the ISG formulations demonstrated a higher density value than that of water; thus, they showed a tendency to be embedded in the crevicular pocket without being lost from the crevicular fluid because they could sink into this.

The borneol-based ISGs possessed a higher viscosity value because of the strong hydrophilic interaction between the borneol solvent and the diminished amount of solvent, as reported previously [18]. The viscosity of ISG solutions containing triacetin dissolved in NMP and DMSO was in the range of 5.55–9.67 and 5.92–10.43 cPs, respectively. Their viscosities were gradually but significantly increased (*p* < 0.05) with the increasing amount of triacetin (Table 1), while the viscosity of triacetin was 25.53 cPs. ISGs in DMSO had a slightly higher viscosity than those in NMP because DMSO is more viscous than NMP. The viscosity of each formulation in this study was <20 cPs, indicating the modest viscosity for a facile injection via needle when administered into the crevicular pocket [36]. These basic physicochemical characteristics were applied to explain the results of contact angles, surface tension, and injectability, including the phenomenon of solvent exchange at the interface of each formulation after exposure to the aqueous phase.

The presence of borneol in the formulation led to a lower surface tension of the borneol-based ISG compared with the borneol-free formulation (Table 1). This can be attributed to the steric hindrance effect caused by borneol in the mixture. Additionally, the 40% borneol interfered with the intermolecular forces of the solvent and increased the free volume space, as previously reported [18,37]. On the other hand, the addition of triacetin had a reducing impact on the surface tension of the mixture. This can be attributed to the similar surface tension values of triacetin and the solvents (NMP and DMSO) present in the mixture.

### 2.2. The Wettability of ISG in Different Types of Surface

**Figure 1:**
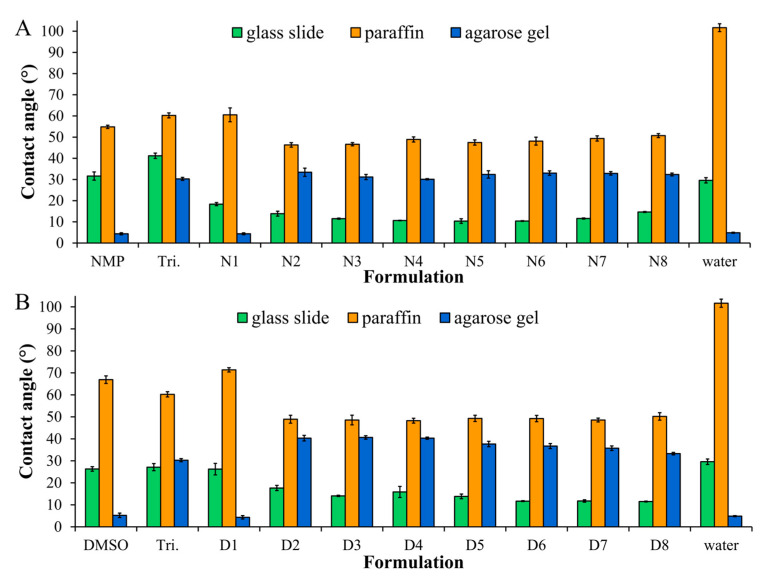
The contact angle of different formulations using NMP (**A**) or DMSO (**B**) as the solvent (n = 3).

Wettability is defined as the ability of a fluid to maintain contact with a surface, and it reflects the nature of the hydrophilic or hydrophobic interaction with the target surface [38]. As previously reported, wettability is studied through measurements of contact angles of ISG on different surfaces. Furthermore, gel formation is also observed from the change in the ISG contact angle on a water-containing surface, such as an agarose gel [18]. Herein, a glass slide, paraffin-coated glass slide, and agarose gel containing water were used as the test surfaces. The contact angles of the borneol-based ISGs on the glass slide were lower than those of the solvents (NMP or DMSO) (Figure 1). The interference of borneol molecules decreased the cohesive force of the solvent molecules and diminished the interactions between DMSO and the glass slide [18,37]. In contrast, when comparing borneol-based ISGs in DMSO and NMP, the DMSO-based gels demonstrated a higher contact angle. This is attributed to the higher viscosity of DMSO, which hindered the spreading of droplets on the surface. Furthermore, the reduction in contact angles on the paraffin-coated glass slide in the presence of borneol indicated a significant hydrophobic interaction between borneol molecules (log K_o/w_ of 2.85) and the hydrophobic surface, such as paraffin [18,37]. Nevertheless, the presence of triacetin in the formulation did not significantly influence the contact angle observed between the glass slide and paraffin.

The contact angle was investigated on an agarose gel to mimic the crevicular pocket condition and to assess the behavior of ISG transformation from liquid to gel after exposure to water in the agarose gel surface. The contact angle significantly increased in borneol-based formulations, but not in the case of triacetin-loaded ISGs (Figure 1). The higher viscosity of borneol-based ISG could retard the spreading of droplets on the surfaces [39]. The increased contact angle reflected the instantaneous phase transition from the solution to the gel of the ISG formulations. No contact angle values were >45°, indicating that the formulation had favorable wettability and was likely to adhere to the gingival surface when administered in the crevicular pocket. In comparison, ISGs in DMSO exhibited higher contact angles than ISGs in NMP due to the higher viscosity of DMSO.

### 2.3. Injectability and Mechanical Properties

**Figure 2:**
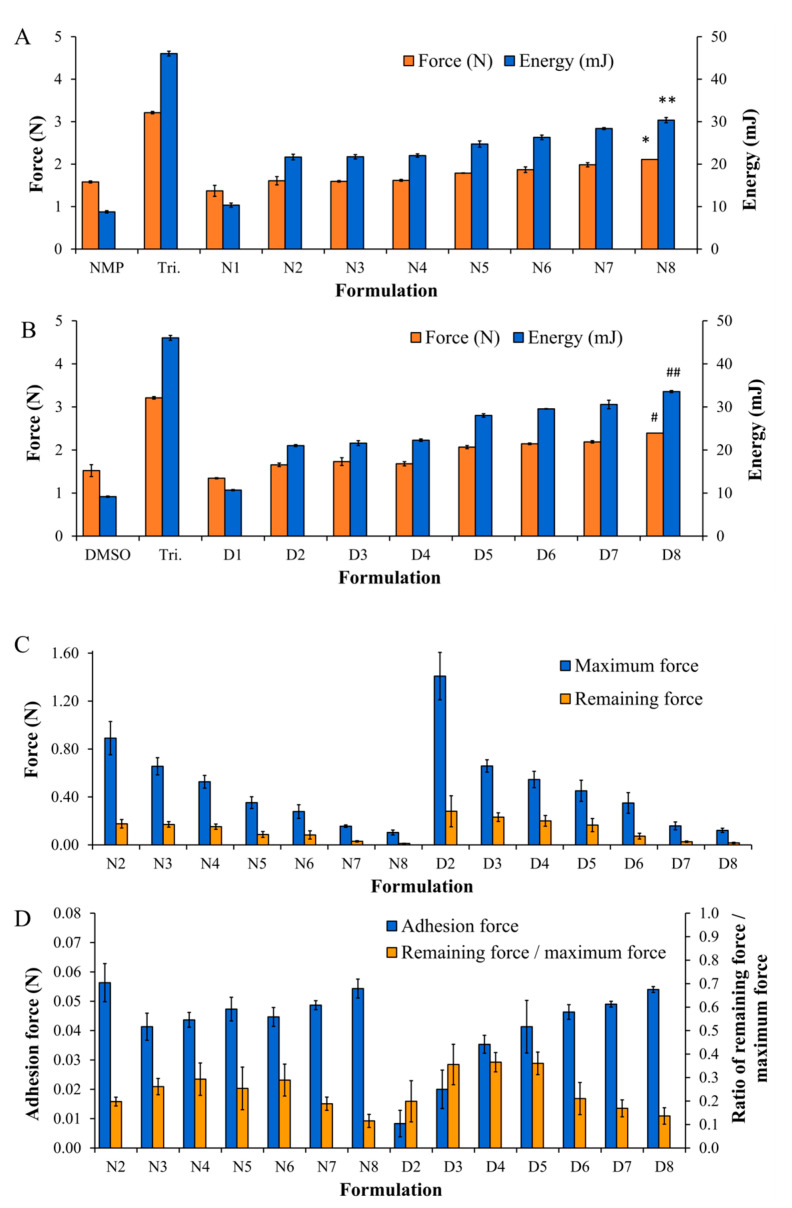
Force and energy for expelling syringes of different formulations using NMP (**A**) or DMSO (**B**) as the solvent (n = 3). Effects of mechanical properties on (**C**) the maximum and remaining force, (**D**) adhesion force and mechanical properties of different formulations (n = 3). Tri, triacetin. *^,^** *p* < 0.05, compared with N2, ^#,##^ *p* < 0.05 compared with D2.

The force and energy required to inject fluid from a syringe via the needle are measured in terms of injectability [40]. Borneol addition enhanced both the maximum force and energy required for the injection, while the triacetin addition slightly increased these. Notably, the maximum force and energy of N8 and D8 were significantly higher (*p* < 0.05) than those of N2 and D2, respectively. However, minimal energy was required for injections of formulations containing different triacetin amounts of both the NMP and DMSO series (21.02–33.56 mJ; Figure 2A,B). The energy used for the injection of these ISG solutions was similar to that of polymeric ISGs containing 5–20% *w/w* ethyl cellulose (21.73–41.98 mJ), 15–30% *w/w* bleach shellac (11.73–27.69 mJ), and 15–35% *w/w* Eudragit RS (8.04–23.99 mJ) [41]. The maximal injection force for all ISGs in this investigation was <10 N, signifying the simplicity of injection and acceptability for injection, including reduced discomfort at the injection site [42]. Consequently, all triacetin-mixed ISGs were acceptable and suitable for use as an injectable drug delivery system, with convenient administration and satisfactory patient comfort/compliance [40,42].

The gels obtained after phase transformation were tested for their mechanical properties. To obtain the gel from the ISG solution, each formulation was inserted into the hole in an agarose gel and left for complete solvent exchange and gel formation. The sturdiness of the gel is reflected in terms of maximum compressive resistance applied to the gel, and the proportions of the remaining force and maximum force also describe the properties of matrices [43]. Both maximum penetration force and remaining force decreased with increasing triacetin ratio, implying the gel sturdiness was also lessened (Figure 2C). Therefore, a high proportion of triacetin in the formulation would be unsuitable for the application of ISG for crevicular pocket drug delivery because of the low gel firmness. Furthermore, other factors such as the rate of the gel formation and the morphology of the gel formation should be considered as well. The adhesion force of each formulation was low and did not considerably differ (Figure 2D). Consequently, the presence of triacetin did not substantially affect the adhesive force of borneol-based ISG. By comparison, the maximum adhesion force was similar to or slightly lower than that of resin-based ISGs containing 35% *w/w* propolis, 35% *w/w* benzoin, or 35% *w/w* rosin (0.24, 0.14, and 0.04 N, respectively) [44]. The ratio of the remaining force and maximum penetration forces was calculated to quantify the deformation properties (elastic and plastic deformation characteristics); the high value of this ratio (close to 1.00) indicates a high elasticity, whereas a low value reflects high plasticity [43]. Herein, the ratio for all formulations was between 0.12 to 0.37, and all obtained matrices, therefore, exhibited plastic deformation properties (Figure 2D). This implied that they were persistent and suitable to deform in a desirable manner in the shape and size of the crevicular pockets [43]. The ratio of other polymer-based in situ forming implants such as poly (D,L-lactic-co-glycolic acid) mixed with a plasticizer (acetyltributyl citrate or dibutyl sebacate) was <0.15 and demonstrated favorable plastic deformability. Additionally, this ratio of the commercial product Parocline*^®^* (Sunstar, Levallois-Perret, France) (a dental gel containing minocycline (2%), hydroxyethyl cellulose, magnesium chloride, Eudragit RS, triacetin, and glycerol) has been reported as close to 0.4 [43].

### 2.4. Morphology of Gel Formation

**Figure 3:**
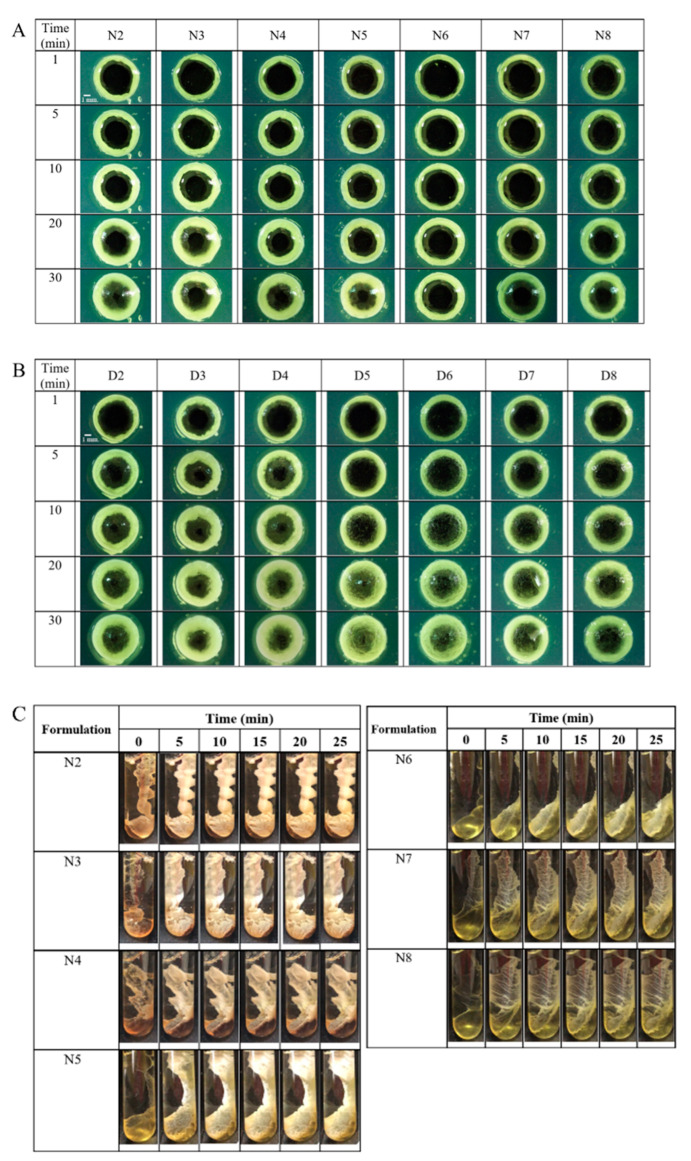
Morphology of gel formation after different formulations using NMP (**A**) or DMSO (**B**) as a solvent filling in an agarose hole containing PBS pH 6.8 under a stereo microscope at 4× magnification. (**C**) Gel formation behavior after the injection of different formulations through a 27-gauge needle into PBS pH 6.8.

The macroscopic gel change of ISG was observed under a stereomicroscope to investigate gel morphology and formation rates. When the formulation was instilled into the agarose hole, the water from the agarose immediately diffused into the formulation, transforming the borneol solution to an opaque white gel owing to the phase inversion of borneol. The NMP in the formulation diffused into the agarose because of its affinity with water, and this process occurred as a solvent exchange with the mutual invasion of the water phase into the ISG, thereby inducing phase transformation of ISG. The gel formation started at the interface between the ISG and the agarose gel. The mass and thickness of the gel continuously increased over time from the interface to the center of the agarose hole, as shown in Figure 3A,B. The thickness of the cloudy gel increased slowly with time with the increase in the ratio of triacetin in the formulation because triacetin retarded the solvent exchange of ISG [29,45]. The borneol gel-formed ISGs using NMP as the solvent were tightly clustered. The gel from the series possessed a loosely clustered appearance, which was observed in the center of the agarose hole 30 min after the formulation was exposed to the aqueous phase of the agarose gel. Moreover, the matrices from both series of ISGs were crystal-like in appearance because of the borneol phase inversion [22]. High gel firmness contributes to controlled drug release because of the entrapment of the drug in the gel, whereas the absence of the sufficient gel firmness may not hold the drug in the gel [22]. Additionally, NMP has a favorable safety profile and has been used as a solvent for ISG in many pharmaceutical commercial products for periodontitis treatment [14,46,47,48]. The European Commission Scientific Committee on Consumer Safety concluded that NMP has low acute toxicity through oral, dermal, and inhalation routes of administration [49,50]. As a result, formulations utilizing NMP as a solvent exhibit favorable properties that make them suitable for being developed into ISG formulations for the treatment of periodontitis. Thus, these formulations were further evaluated in subsequent testing.

ISGs using NMP as a solvent were assessed in gel formation studies to determine how triacetin incorporation affects gel formation. First, a liquid ISG sample was introduced into a test tube containing phosphate-buffered saline (PBS), pH 6.8, to simulate gel formation when injecting the formulation into the crevicular fluid, and the obtained gel formation is shown in Figure 3C. The ISG solution abruptly transformed to a gel state after initially contacting PBS pH 6.8 and progressively converted to a cloudy gel-like matrix with phase inversion, because the aqueous insolubility of borneol induced a sudden phase separation [18,21]. This rapid phase inversion occurred from the solvent exchange between NMP of ISGs and water from the environment [18,51]. Although increasing the amount of triacetin in the ISGs was expected to help retard the drug release, ISGs containing high amounts of triacetin tended to slowly transform compared with those containing lower amounts. Notably, N5, N6, and N8 failed to transform into a gel-like matrix within 25 min, and a soft-like gel appeared instead. This delayed gel formation may retard the efficiency of drug entrapment of the borneol gel and possibly cause failure of extended drug release [18,22]. Conversely, ISG with low triacetin loading such as N3 underwent a too rapid phase inversion into gel that did not enable control of drug liberation. An acceptable gel transformation occurred with N4 because appropriate triacetin loading would enable extended release of the drug, and this formulation was selected for further investigations.

### 2.5. Microscopic Changes in Gel Formation

**Figure 4:**
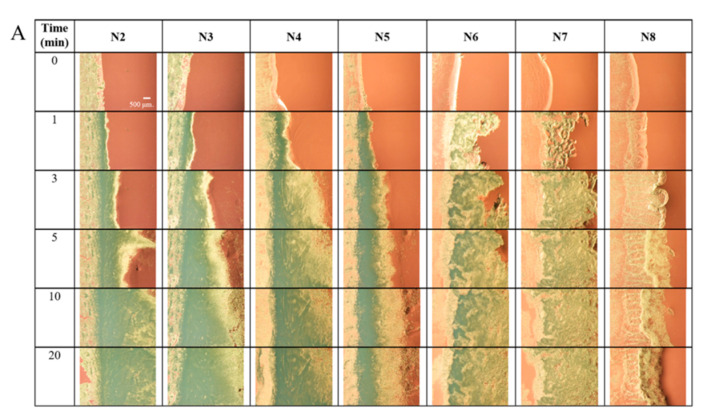

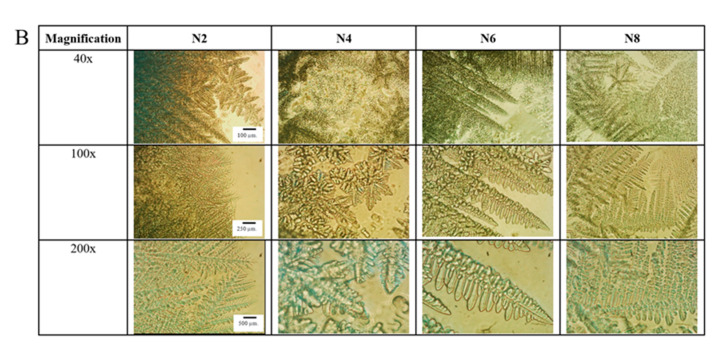
(**A**) Microscopic changes in gel formation at the interface between the formulation and water after contact with PBS pH 6.8 under a stereomicroscope (magnification of 8×) and (**B**) their morphology under an inverted microscope (magnification of 40×, 100×, and 200×).

To gain more understanding of borneol gel formation, microscopic observation of the interface was undertaken after contact with ISG formulation with the agarose gel rim under a stereomicroscope, as shown in Figure 4A. A gel formed at the interface and expanded over time, especially into the formulation portion (right side). The gel originated from the beginning of the borneol phase separation. Typically, after the prepared ISG solution makes contact with the crevicular fluid in the crevicular pocket, the organic solvent from the formulation diffuses into the environment while the water molecules diffuse into the formulation in a process known as solvent exchange. The cloudy white borneol gel continuously formed because of the phase separation of water-insoluble borneol. Drug molecules are subsequently enclosed in the gel and are then progressively released over time [16,17,18]. In the early phase, the rate of solvent exchange usually occurs rapidly and gradually reduces over time as the reaction reaches equilibrium as a result of the thermodynamic theory [52,53]. A higher borneol content was found nearby the interface area and gradually decreased with increasing the distance from the interface [54]. A black opaque cluster in several parts caused by gel formation was deposited in many layers in the gel (Figure 4A). During this process, a solid precipitate was formed, which had a dense structure that prevented the passage of light through it when observed under a stereomicroscope. The high triacetin content in the formulation slowed the diffusion of solvents, causing slow gel formation and a noticeably lower gel firmness of ISG, as shown in the results of N6, N7, and N8. Conversely, the low triacetin-loaded ISG transformed into a gel with a high firmness with dark clusters, as observed under the microscope. Noticeably, dense gel clusters tended to form at the interface, whereas loosely clustered gel formed further from the interface area. Additionally, the gel of high triacetin-loaded ISG exhibited reduced gel aggregation attributed to its interference against borneol gel formation.

The morphology of the obtained gel after the complete solvent exchange was observed under an inverted microscope (Figure 4B). The ISG without triacetin (N2) formed small grain-shaped borneol crystals with a continuously intricate gel. However, the presence of triacetin and a high viscosity extended the induction time required for gel growth [55]. In addition, the triacetin-induced hydrophobicity in the system reduced the solvent exchange of ISG and the late phase inversion of borneol [56]. Consequently, a borneol gel with large grain-shaped and translucent crystals appeared in triacetin-loaded ISG formulations. At the interface, the nucleation process occurred because of the high borneol content adjacent to the aqueous phase of PBS. Consequently, the crystallization and aggregation of the crystal progressed according to thermodynamics [57,58]. These ISG nucleation and crystallization processes have been described using ibuprofen and fatty acid as gelling agents [17,54]. The examination of the topography of borneol matrices using scanning electron microscopy (SEM) was not conducted in this study because borneol sublimation occurred under vacuum conditions at a high voltage [22].

This experiment was designed to observe the beginning of gel formation and thereby to understand gel formation at the macroscopic level. The characteristic borneol transformation observed at the microscopic level helped to explain the gel formation of the formulation after contact with the aqueous phase and the continuous progressive gel growth observed at the macroscopic level corresponding with the result in Section 2.4. Moreover, the morphology of the formed gel was beneficial for the elucidation of the rate and pattern of drug release for each ISG, which will be discussed in the next section.

### 2.6. Water-Induced Gel Formation Sensitivity

Water titration was used to investigate the water sensitivity or aqueous tolerance of the prepared ISG by measuring the amount of water needed to induce the phase inversion of borneol [17,36]. These data indicate the water sensitivity of the ISG solution and the ease of phase inversion. A low water loading capacity implied that even a minimal amount of water could induce phase inversion. In this experiment, the percentage of water capacities of N2, N4, N6, and N8 were 18.87% ± 0.73%, 20.18% ± 0.77%, 22.99% ± 0.32%, and 26.17% ± 0.40% *v*/*v*, respectively. This value increased with the increase in the triacetin amount in the formulation because of the high hydrophobicity and viscosity of triacetin [59]. Normally, the increased viscosity causes difficulty in gel formation. For example, the bleached shellac-based ISG solution using 2-pyrrolidinone (PYR) as a solvent usually presents a higher water resistance or water capacity due to the high viscosity of PYR [36,59]. The high viscosity of an ISG solution can also retard the solvent exchange and reduce the rate of gel formation [22,36]. Hydrophobic solvents such as triacetin are recognized to produce a slow-forming phase inversion because of the limited water solubility (around 7%) [29]. Triacetin has a low water affinity [33], and the ISG solution containing triacetin was more viscous [32], which diminished the solvent exchange rate and reduced the borneol phase inversion [59]. Consequently, more water was required to induce borneol gel formation, signifying a low susceptibility to water induction. Moreover, the diminished amount of hydrophilic solvent such as NMP in the formulation decreased the overall hydrophobicity of the formulation. In comparison, triacetin (log P = 0.25) was more hydrophobic than NMP (log P = −0.38) [29,60], whereas borneol acted as a nonpolar substance (log P = 2.85) [21]. Consequently, borneol behaved more similarly to triacetin in terms of hydrophobicity than to NMP because of their similar polarities; thus, the water capacity of N8 was the highest among the formulations. The hydrophobic ibuprofen-based ISG dissolved in DMSO exhibited a lower water capacity than that using NMP as the solvent because the log P of DMSO (−1.35) was lower than that of NMP (−0.38); therefore, easier ibuprofen phase inversion occurred with DMSO than with NMP [17]. These results confirm and correspond to the gel formation of borneol-based ISGs in both morphology and microscopic changes. The hydrophobicity, log P value, and viscosity were the crucial factors affecting the sensitivity of water-induced gel formation [17,36,59]. Although there is no specification indicating the optimum value for the water capacity of ISG, a high value may be reflected in slow gel formation and ultimately lead to a burst drug release from ISG. Conversely, low values may pose a problem during pre-formulation as gel formation may be induced by air humidity and before injection of the ISG into the crevicular pocket or too rapid gel formation before the formulation fits the crevicular pocket geometry [18]. Typically, the ISG for periodontal pocket drug delivery is a freshly prepared dosage form [15]. After being freshly prepared, the Atridox^®^ (Tolmar Inc., Fort Colin, CO, USA) product intended for the treatment of periodontitis takes the form of a viscous liquid, containing a concentration of 10% doxycycline hyclate [15,18]. Once it comes into contact with the fluid in the periodontal pocket, the liquid transforms into a solid form, enabling a controlled release of the drug over a span of 7 days [20]. As a result, any potential instability of the developed ISG should not be noticeable.

### 2.7. In Vitro Drug Release

**Figure 5:**
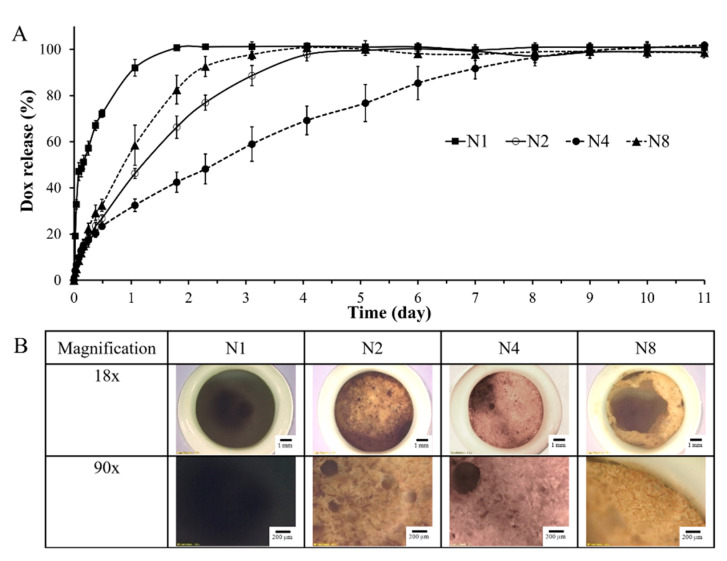
(**A**) Dox release profiles of Dox-loaded ISG formulation in PBS pH 6.8 (n = 3). (**B**) The gel of their formulation after completed Dox release in a porcelain cup under a stereomicroscope (magnification of 2.6×, and 13.0×).

One possible approach to prolong drug release is to include a hydrophobic component, such as triacetin, in the formulation [29,30]. However, the appropriate content of triacetin must be evaluated to ensure the correct proportion required for extended drug release. The gel formation in both microscopic and macroscopic levels showed that the duration of this increased with increasing triacetin content, which was also supported by the data from the sensitivity of water-induced gel formation. Consequently, N4 with a low triacetin content (5% *w/w*) and N8 with a high triacetin content (25% *w/w*) were selected as the testing formulations, along with N2 as an original formulation with borneol-based ISG without triacetin; the control group was Dox solution in NMP (N1). The porcelain cup or membrane-less method, where the sample was added into a tiny porcelain cup and placed into 100 mL of PBS buffer, was selected in this study to simulate drug administration into the crevicular pocket [18]. This method closely simulated the conditions present in the periodontal pocket, as the ISG formulation came into direct contact with the crevicular fluid from the surrounding environment. Moreover, the gel formation was observed to occur promptly upon exposure to the release medium [18,40,61]. The release patterns of Dox from test ISGs in PBS pH 6.8 using this method are presented in Figure 5A.

During the first 6 h, approximately 14.93% of Dox was released from N8, N2, and N4. Meanwhile, N1 without borneol and triacetin rapidly released up to 51.20% of Dox as a lack of gel formation did not entrap the drug [17,18]. The release of Dox continued from N1 to reach 90% within 1 day. N2 transformed to a gel-like matrix after contact with the release medium and its release reached 80% after 2.5 days. The hydrophobic borneol gel enabled prolonged Dox release over time [18]. Other hydrophobic matrices, such as 25% Eudragit L, 15% Eudragit RS, 30% blended shellac, and 40% ibuprofen, could sustain Dox release for >7 days [16,17,62,63]. Vancomycin HCl-loaded borneol-based ISG has been shown to act as a potentially effective local antimicrobial drug delivery system for periodontitis treatment through crevicular pocket injection [18]. A 40% borneol formulation was successful in controlling the drug release; nevertheless, potential novel techniques are being researched for targets of prolonged drug release. Typically, a drug is quickly released from its deposit on the gel surface at the initial state and then it is gradually liberated over time through a longer diffusion route [18]. The long path length with high tortuosity and low porosity of the gradually formed borneol gel over time is crucial for decreasing the Dox release rate [64]. Practically, burst drug release should be minimized for more efficient control of drug release from drug delivery systems, including injectable ISG systems. Reducing the hydrophilicity of the system by incorporating a hydrophobic substance into the ISG system is a useful technique for solving this issue [56]. For the mixed solvent systems, a hydrophilic solvent (e.g., NMP) promotes a rapid phase inversion causing a rapid diffusion of the solvent and sudden solid-like gel formation, but a hydrophobic solvent (e.g., triacetin) slows the phase inversion to limit solvent diffusion and subsequent drug release [29,30,65]. Thus, 5% triacetin was added into the ISGs to modify the drug release. Dox release was subsequently extended by the addition of 5% triacetin into the ISGs, as shown in the release profile of N4, which completely transformed into a gel-like matrix within 2 h. 

Interestingly, the incorporation of high levels of triacetin, up to 25%, in the ISG formulation resulted in delayed gel formation. This can be attributed to the hydrophobic nature of triacetin, which initiated a potentially slower solvent exchange process. [29,30,45]. Therefore, the gel formation was difficult to achieve and drug retention in the gel was not practicable [30]. Consequently, the release profile of N8 was faster than that of N2, but still slower than that of N1, and it was impractical for controlled drug release. Triacetin prolonged the drug release because of the polymer hydrophobicity; however, the effect was greater with a hydrophobic polymer and lesser with a hydrophilic polymer [30]. To illustrate, the utilization of triacetin as a cosolvent in the ISG formulation was found to be more effective at reducing the burst release with the hydrophobic poly(D,L-lactide) gel. In contrast, its effectiveness was relatively lower when used with the relatively hydrophilic Resomer*^®^* RG 502, poly(D,L-lactide-co-glycolide) gel [30]. Thus, the optimum concentration of triacetin as a cosolvent for prolonged drug release depended on the type of gelling agent [30]. The high content of triacetin combined with a hydrophilic solvent may not be guaranteed to prolong the release of a drug. Consequently, N4 containing 40% borneol and 5% triacetin was effective at prolonging the drug release for 10 days. This minimized the burst Dox release and controlled the drug release continuously over time. Atridox*^®^*, a commercial ISG featuring DH and 33.03% poly(D,L-lactide) as a gelling agent, controls drug release for 7 days after administration into the crevicular pocket [20].

The ideal solvents used for ISG systems should be safe to be injected into the body, as well as being biocompatible and biodegradable [33]. ISG systems most commonly consist of NMP as a solvent, which is preferentially used because of its safety and pharmaceutical precedence over other solvents [65]. NMP is known as a solubilizing excipient used in parenteral and oral medications [50]. For the treatment of periodontitis, NMP has favorable safety data and has been used as a solvent of ISG in many pharmaceutical commercial products such as Atridox*^®^*, Periocline*^®^*, and Arestin*^®^* (OraPharma, Horsham, PA, USA) [14,46,47,48]. According to the EU Scientific Committee on Consumer Safety, NMP has a low acute toxicity through oral, dermal, and inhalation routes of administration [49,50]. Additionally, NMP has been classified as Class 2 based on ICH guidelines and is recommended for use within low concentrations in pharmaceutical products [33]. The median lethal doses of NMP are >2 mL/kg and are considered to be safe for use in ISGs [66]. Triacetin is a nontoxic edible fatty acid and is generally recognized as safe [32]. Furthermore, triacetin has been approved as a food additive by the US FDA and has been used as a parenteral nutrient [32].

After the completion of drug release experiments, the remnant of each formulation was removed from PBS pH 6.8 and the excess water was removed. The surface morphology of the borneol gel after being transformed into a gel-like matrix soaked in PBS for 11 days was investigated as shown in Figure 5B. Nothing was found in the cup containing N1 owing to the lack of borneol in the formulation, whereas spongy solid matrices with amber coloring were found in the cups of N2, N4, and N8, which were remnants from the water-insoluble borneol matrices. At a high content of triacetin, the phase inversion was slow, resulting in the gel being difficult to form and rarely occurring, referring to all of the reasons mentioned above. Hence, the gel from N8 usually appeared to surround the cup inside, as seen from the cross-sectional image, but it did not form in the middle of the cup. The addition of triacetin seemed to increase the gel porosity [30]. The cross-sectional images of poly(D,L-lactide) matrices in the DMSO/triacetin mixed solvent and their cross-sectional morphologies as a function of time have been investigated previously [30]. The gel cross section from formulations using only DMSO as the solvent exhibited much denser structures than that of the gel from the solvents consisting of triacetin. Furthermore, it was observed that the gel formed from a higher amount of triacetin in the solvent system exhibited a more porous structure, and this porosity was more pronounced [30]. In comparison, the gel from N4 was more porous than that of N2. Normally, the efficiency of controlling drug release from ISG corresponds to the topography and it is usually investigated with SEM [17]. The examination of topography under SEM was not achieved in this investigation [22] because of the sublimation of borneol. Consequently, stereomicroscope observation was applied in this work.

### 2.8. Drug Release Kinetics

The estimated parameters from drug release profiles fitting different mathematical equations are shown in Table 2. Mathematical equations, including zero-order, first-order, Higuchi’s, Korsmeyer–Peppas’s, Hixon’s, Hofenberg’s, and Peppas–Sahlin’s equations, were applied. The release profile of Dox from N1 matched with the Korsmeyer–Peppas (R^2^ = 0.9956, AIC = 17.5847, MSC = 4.5607) and the Peppas–Sahlin models (R^2^ = 0.9979, AIC = 21.2822, MSC = 5.1775). Moreover, the drug release was governed by diffusion, and the diffusion rate corresponded to the drug concentration in the formulation at that time. This release kinetic is described as the Fickian diffusion model and is characterized by diffusivity because the n value from the Korsmeyer–Peppas model was 0.318 (<0.45) [67]. Additionally, the k_1_ value obtained from the Peppas–Sahlin equation remained higher than the k_2_ value (k_1_ = 8.630 and k_2_ = −0.048), which supports the hypothesis that the release mechanism from Fickian diffusion was more prominent than that of Case II relaxation [67]. For N2, this also matched with the Korsmeyer–Peppas model (R^2^ = 0.9984, AIC = 40.7684, MSC = 6.0316) and Peppas–Sahlin model (R^2^ = 0.9984, AIC = 42.7074, MSC = 5.8825). The kinetic release of N2 was described as non-Fickian or anomalous transport because the n value from the Korsmeyer–Peppas’ model was 0.629 (0.5 < n < 1). This drug-release mechanism was governed by diffusion and swelling of the gel. The diffusion and swelling rates were comparable.

The k value from the Peppas–Sahlin model was used to describe the mechanism of diffusion and swelling in detail. In this case, k_2_ was more dominant than k_1_ (k_1_ = −0.157 and k_2_ = 0.518), and the swelling or erosion mechanism from the borneol gel was subsequently more prominent than the diffusion mechanism. The release profile of N4, comprising 5% triacetin and 40% borneol, also matched with the Korsmeyer–Peppas model (R^2^ = 0.9974, AIC = 57.9719, MSC = 5.6128), while the n value was 0.518. The Peppas–Sahlin model of N4 (R^2^ = 0.9987, AIC = 48.5514, MSC = 6.2015) showed that k_1_ was more dominant than k_2_ (k_1_ = 1.291 and k_2_ = 0.026). Thus, the addition of a hydrophobic substance (e.g., triacetin) into the ISG produced a gel with the Fickian diffusion mechanism of drug release. Moreover, the release profile of N8 matched the Korsmeyer–Peppas (R^2^ = 0.9985, AIC = 10.5336, MSC = 5.7222) and Peppas–Sahlin models (R^2^ = 0.9986, AIC = 12.3015, MSC = 5.3686). However, the release kinetics were non-Fickian or anomalous transport with n = 0.679. In comparison, the diffusion release mechanism of N8 appeared to be less pronounced than that of N4, because the k_1_ value of N8 was lower than that of N4. However, the release mechanism of N8 caused by erosion or swelling was significant because of the increased k_2_ value compared with that of N4. However, the diffusion mechanism was a dominant drug-release mechanism of N8 when considering both k_1_ and k_2_ values due to the predominant k_1_ (k_1_ = 0.652 and k_2_ = 0.191).

### 2.9. Antimicrobial Activities

Periodontitis is classified as an oral health disease, and a variety of pathogens can cause the disease [2,68]. Microbial proliferation in the crevicular pocket produces periodontal inflammation and damage, which accelerates attachment loss and loss of bone around the teeth [68]. The predominant pathogens that are most common in many patients with periodontitis are *A. actinomycetemcomitans* and *P. gingivalis* [4], and they were thus selected for testing in this study. In the case of a patient where the environment surrounding periodontitis is disturbed, several commensal bacteria such as Actinomyces and certain *Streptococcus* and *Staphylococcus* spp., can invade the periodontal structures and cause opportunistic infections [68]. Furthermore, *S. aureus* can be isolated from the periodontal pockets of individuals with aggressive periodontitis, while *E. coli* has been associated with patients suffering from periodontitis [6]. Thus, *S. aureus* and *E. coli* were chosen as Gram-positive and Gram-negative bacteria, respectively, to assess the antibacterial activity. Furthermore, *Candida* spp. can work in concert with other dominant microbes in periodontitis disease, even though these have not yet been identified as principal periodontal microorganisms [69]. Refractory periodontitis has been related to infection with oral flora such as *C. albicans* [70]. Accordingly, *Candida* spp., including *C. albicans*, *Candida krusei*, *Candida lusitaniae*, and *Candida tropicalis*, were also used for antimicrobial testing. Presently, opportunistic infection is a major issue, particularly hospital-acquired opportunistic infections. Patients with severe periodontitis, especially those requiring surgical treatment, were usually infected by nosocomial and opportunistic infections. In the United States, >50% of infections case in the ICU are caused by methicillin-resistant *S. aureus* (MRSA), which is a well-known opportunistic pathogen [71]. Therefore, MRSA was also selected as a microbe in this test.

**Figure 6:**
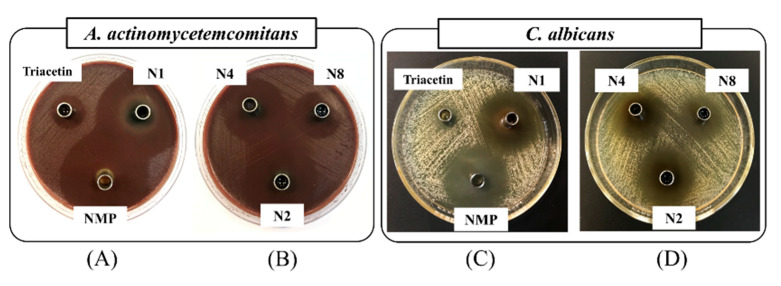
Photographs of the inhibition zone of ISG formulations, NMP, and triacetin against *A. actinomycetemcomitans* (**A**,**B**) and *C. albicans* (**C**,**D**) (n = 3).

The selected formulations (N1, N2, N4, and N8) were chosen for antimicrobial testing; NMP and triacetin were tested as the control groups. This experiment was performed following the cup agar diffusion method, as previously reported [17,18]. The inhibition zone diameters of the test formulations against *P. gingivalis*, *A. actinomycetemcomitans*, MRSA, *S. aureus*, *E. coli*, *C. albicans*, *C. krusei*, *C. lusitaniae*, and *C. tropicalis* are summarized in Table 3. Moreover, the inhibition zones of the formulations against *A. actinomycetemcomitans* and *C. albicans* are displayed in Figure 6. Interestingly, the clear zones observed in the formulations containing borneol (N2, N4, and N8) were smaller compared with N1 because the borneol gel retarded Dox diffusion into the inoculated agar [18]. Typically, ISGs containing other water-insoluble gel formations such as Eudragit L and zein exhibit a decreased size in the clear zone due to their restricted penetration of the bioactive agent [16,72]. Furthermore, the addition of triacetin in the formulation diminished the Dox diffusion. The ability of the drug movement also depends on the concentration of triacetin. The drug diffusion into the agarose gel was retarded when the formulation contained a higher amount of triacetin because of the hydrophobicity of triacetin. All of the selected formulations (N1, N2, N4, and N8) exhibited excellent antimicrobial activities against *P. gingivalis*, and their inhibition effects were significantly higher than those of NMP (*p* < 0.05) because Dox in the formulation showed a markedly effective inhibition against *P. gingivalis* above the MIC value [73].

However, NMP was effective against *P. gingivalis* because NMP could solubilize the lipids in the cell membrane and promote the leakage of microbial cell membranes [19]. Thus, the antimicrobial effects of both NMP and Dox encouraged the effectiveness of antimicrobial activities. Although the antimicrobial effectiveness of N4 was rather decreased because of the addition of borneol and triacetin, the clear zone of N4 remained significantly larger than that of NMP (*p* < 0.05). All Dox-loaded formulations presented antimicrobial activities against *A. actinomycetemcomitans* because Dox is highly effective against this anaerobic bacterium [74]. NMP also displayed a notable antimicrobial activity against *A. actinomycetemcomitans,* as reported previously [17]; thus, while the inhibition zone diameter of NMP was rather high, this was still lower than that of N1 (*p* < 0.05). The addition of borneol reduced the inhibition zone diameter as its gel slowed the diffusion of solvent and drug molecules. However, the inhibition against *A. actinomycetemcomitans* by N4 was still considered effective and remained satisfactory. Dox showed significant antimicrobial effectiveness against MRSA, *S. aureus*, and *E. coli* (*p* < 0.05) when compared with that of NMP [17,19]. Additionally, borneol can inhibit bacteria because the chiral carbons have antiadhesive properties for bacterial cells and interfere with bacterial colonization [75]. Despite N4 containing both borneol and triacetin, the clear zone observed for this formulation was notably larger compared with NMP (*p* < 0.05), and showed an efficient antimicrobial activity against both Gram-negative and Gram-positive bacteria, including drug-resistant bacteria. Although Dox does not inhibit yeast, NMP as a solvent in formulation efficiently inhibited these fungi; thus, the inhibition zone against *Candida* spp., including *C. albicans*, *C. krusei*, *C. lusitaniae*, and *C. tropicalis* between NMP and N1 was not significantly different [17,76]. Although, borneol has an antifungal activity against *Candida* spp. by promoting the generation of lipid peroxides, a solid-like borneol gel could retard the solvent diffusion on agarose [77]. As aforementioned, borneol was transformed into a gel-like matrix and retarded the diffusion of NMP and triacetin into an inoculated agar; thus, the clear zone of borneol-containing ISG appeared smaller than that of N1. However, N4 still exhibited an efficient antifungal efficacy because of the antifungal activities of NMP and triacetin [76,78].

The MICs of Dox against *P. gingivalis*, *A. actinomycetemcomitans*, MRSA, *S. aureus*, and *E. coli* are 0.125, 0.21, 8.00, 0.28, and 1.54 mg/L, respectively [73,74,79,80]. Normally, patients with periodontitis often have 0.5 to 1.0 µL of gingival crevicular fluid in their crevicular pockets [81]. Assuming that 1.0 µL of the formulation was administered into the pocket, the contents of 5% Dox released from N4 at 1 h, 1 day, and 5 days were 2500, 11,500, and 38,000 mg/L, respectively. These values are substantially higher than the MIC values against all of the test microbes and provoked antimicrobial efficacy initially from the first hour of administration. In conclusion, our prepared Dox-loaded ISG containing borneol and triacetin exhibited an effective antimicrobial activity for periodontitis treatment.

## 3. Conclusions

Triacetin was successfully employed as an additive hydrophobic solvent of Dox-loaded 40% borneol-based ISG. The prepared ISGs appeared as homogeneous transparent solutions, and their density and viscosity gradually decreased with the addition of triacetin when their viscosity was <12 cPs. However, triacetin slightly influenced the surface tension of ISG. Increasing the triacetin proportion enhanced the injection force and energy needed to expel the ISG from the needle; nevertheless, the force required was <10 N, demonstrating the convenience of administration by injection into the crevicular pocket. Once ISGs contacted the aqueous phase, they transformed into a gel-like matrix by solvent exchange and phase inversion. The gel formed from ISGs exhibited favorable wettability with a low contact angle (<45°) and presented plastic deformation. NMP was considered as a proper main solvent of ISG because the obtained gel showed increased firmness. High triacetin (25%)-loaded ISG tended to slow solvent diffusion, gel formation, and gel firmness, but exhibited less water sensitivity. Conversely, ISG with a low triacetin concentration (2.5%) exhibited an overly rapid phase inversion and a rapid Dox release within 4 days. The most appropriate ISG containing 5% triacetin showed the longest Dox release for 10 days with Fickian diffusion. Moreover, this formulation presented effective antimicrobial activities against various microbes, including periodontitis pathogens such as *P. gingivalis* and *A. actinomycetemcomitans*. Therefore, Dox-loaded 40% borneol-based ISG with 5% triacetin is a potentially effective local ISG for periodontitis treatment via crevicular pocket injection. When compared with a systemic drug regimen, this developed ISG could provide higher antimicrobial drug concentrations in the subgingival site and decrease the risk of drug resistant microbial populations at non-oral body sites. In addition, it is less invasive when compared with surgical intervention. However, it is the responsibility of the dentist to carry out the application of ISG on the patient. Despite the availability of safety data regarding the medical applications of borneol and triacetin, it is crucial to conduct additional clinical experiments to assess the efficacy and safety of this developed Dox-incorporated borneol-based ISG.

## 4. Materials and Methods

### 4.1. Materials

Dox (lot No. 20071121) was received from Huashu Pharmaceutical Corporation, Shijiazhuang, China. Borneol was purchased from Chareorsukosop herbal shop (Nakhon Pathom, Thailand). DMSO (lot no. 1862992, Fisher Chemical, Loughborough, UK), NMP (lot no. LR00971908, Loba Chemical, Mumbai, India), and triacetin (lot no. A0426392, Acros Organics, Geel, Belgium) were obtained as indicated. Phosphate-buffered saline (PBS) consisted of sodium hydroxide (lot no.121458-0415, QREC, Auckland, New Zealand) and potassium dihydrogen phosphate (lot no. 82804-0711 QREC, Auckland, New Zealand) dissolved in deionized water and adjusted to pH 6.8, and was prepared to mimic the crevicular pocket condition. Agarose (lot no. C7031-17, Vivantis, Selangor Darul Ehsan, Malaysia) and fully refined paraffin wax (lot no. 7119K18, Nippon Seiro, Yamaguchi, Japan) were employed as received. Microorganisms used to assess the antimicrobial activity were *P. gingivalis* ATCC 33277, *A. actinomycetemcomitans* ATCC 29522, MRSA ATCC 43300, *S. aureus* ATCC 25923, *E. coli* ATCC 8739, *C. albicans* ATCC 17100, *C. krusei* TISTR 5259, *C. lusitaniae* TISTR 5156, and *C. tropicalis* TISTR 5306 (Thailand institute of scientific and technological research, Pathum Thani, Thailand). Media were prepared from tryptic soy agar (TSA) (lot no. 3071341), tryptic soy broth (lot no. 9139963), Sabouraud dextrose agar (SDA) (lot no. 9113804), and Sabouraud dextrose broth (SDB) (lot no. 9131072) and were purchased from Becton, Sparks, MD, USA. Sheep blood agar and chocolate agar were purchased from the Department of Medical Science, Ministry of public health, Nonthaburi, Thailand. Deionized water was used in all of the experiments and purified using SG Ultra Clear 2002 water system (SG Water GmbH, Barsbuttel, Germany).

### 4.2. Preparation of ISG

ISG solutions containing Dox 5% as a model drug and borneol 40% *w/w* as a gel forming agent were prepared by dissolving in NMP (formulation code starts with N) and DMSO (formulation code starts with D). Various amounts of triacetin 2.5%, 5%, 10%, 15%, 20%, and 25% *w/w* were dissolved in these ISG solutions (N3, N4, N5, N6, N7, and N8, and D3, D4, D5, D6, D7, and D8, respectively). Dox-loaded borneol-based ISGs without triacetin dissolved in NMP and DMSO were prepared as N2 and D2, respectively. N1 and D1 were Dox loaded in NMP and DMSO, respectively. These ISG solutions were stirred continuously for 3 h until clear solutions were obtained. The components for all formulations are shown in Table 4.

### 4.3. Physicochemical Characterization of ISG

#### 4.3.1. Appearance and Density

The visual characteristics (e.g., color and homogeneity) of the prepared ISG solutions were observed. The density value of the ISG solutions and relative solvents was measured using a pycnometer (Densito 30PX, Mettler Toledo Ltd., Vernon Hills, IL, USA) at 25 °C (n = 3).

#### 4.3.2. Viscosity

The viscosity of all ISG solutions and relative solvents was measured using a viscometer (Brookfield DV-III Ultra, Brookfield Engineering Laboratories Inc., Middleboro, MA, USA) with spindle number 40 at 150 rpm (n = 3). The temperature was set at 25 °C.

#### 4.3.3. Surface Tension, Contact Angle, and Wettability

The surface tension of ISG solutions was evaluated by observing the change in the shape of a pendant drop of the liquid suspended in the air. This assessment was conducted using a goniometer (FTA 1000, First Ten Angstroms, Newark, CA, USA) with a pump out rate of 1.9 μL/s. The measurements were performed three times (n = 3).

To determine the contact angle, the ISG solution was dropped onto various surfaces, including glass slides, paraffin-coated glass slides, and agarose gel (0.6% agarose in PBS pH 6.8). The same goniometer mentioned earlier, with a pump out rate of 1.9 μL/s, was used to measure the contact angle. The images of the droplets on the test surfaces were captured 5 *s* after deposition. The degree of contact angle was then calculated based on these images [18,37]. The collected data from these experiments provided valuable insights into the wettability of the ISG solutions on the different test surfaces.

#### 4.3.4. Injectability and Mechanical Properties

To assess the injectability of the ISG formulation, the force required to expel 1 mL of the formulation through a 27-gauge needle was measured. This measurement served as an indicator of the ease of injection. The compression mode of a texture analyzer (TA.XT plus, Stable Micro Systems, Surrey, UK) was employed to quantify the expelling force. The upper probe of the texture analyzer was positioned directly on the syringe plunger, applying a constant force of 0.1 N and a speed of 1.0 mm/s until the syringe barrel base was reached. This process was repeated three times (n = 3). The maximum force required to expel the formulation through the needle was recorded, and the energy expended during the expulsion process was calculated based on the force–displacement profiles.

The mechanical properties of the obtained gel after complete solvent exchange were determined using the same instrument as above. The agarose gel (0.6% agarose solution in PBS pH 6.8) was poured onto Petri dishes to a 1 cm height. Once the gel had solidified, it was cut with a cylinder cup (7 mm diameter) in the center of the Petri dish. A volume of 150 μL of the ISG sample was added into the agarose hole and left for 72 h for complete the gel formation. A stainless spherical probe (5 mm diameter) of the texture analyzer (TA.XT plus, Stable Micro Systems, Surrey, UK) was gradually pressed into the obtained gel at a constant speed of 0.5 mm/s. At a penetration depth of 1.5 mm, this probe was held for 60 s and then moved backward at a speed of 10 mm/s. The relationship between the applied force and the displacement of the probe was plotted as a function of time and recorded for the maximum penetration force. The remaining force was measured for 60 s after the probe was held, and the adhesion force was the maximum force measured while moving backward (n = 3). The mechanical property was presented in the ratio of the remaining force to the maximum penetration force; where high values imply high elasticity while low values imply high plasticity [43]. All of the formulations were determined in triplicate at 25 °C.

### 4.4. The Evaluation of Gel Formation

#### 4.4.1. The Morphology of Gel Formation

An agarose hole was prepared in the agarose gel as above. Then, the prepared ISG solution of 150 μL was added into the hole. A stereo microscope (SZX10, Olympus Corp., Tokyo, Japan) was used to observe the morphology of gel formation after contact with the aqueous phase from the rim of the agarose gel. Images were recorded at 1, 5, and 30 min with a magnification of 4× after adding the sample into the agarose hole.

#### 4.4.2. Gel Formation after Injection

The prepared ISG solution (1 mL) was injected through a stainless 27-gauge needle into a test tube containing 5 mL of PBS pH 6.8 to investigate the morphology of gel formation after administration via a needle. The images were obtained every 5 min for 25 min after injection into the buffer.

#### 4.4.3. Microscopic Changes in Gel Formation

The origin of gel formation and its expansion at the interface between the formulation and agarose gel was observed under a stereo microscope (SZX10, Olympus Corp., Tokyo, Japan) to investigate the phase inversion phenomena and microscopic changes of the gel. First, the prepared ISG solution (20 μL) was dropped on a glass slide. Then, 20 μL of PBS pH 6.8 was dropped close to the ISG solution and then the gel formation at the interface was recorded 0, 1, 3, 5, 10, and 20 min after the PBS contact with ISG at a magnification of 8×. Additionally, the ISG was dropped close to the agarose gel on a glass slide for investigating the gel formation in detail at the microscopic level under an inverted stereomicroscope (Nikon Eclipse TE2000S, Nikon, Kawasaki, Japan) at magnifications of 40×, 100×, and 200×.

#### 4.4.4. Water-Induced Gel Formation Sensitivity

To determine the minimum amount of water required to induce the phase inversion of the ISG solution into the turbid dispersion, the ISG solution (5 mL) was first added into a 50 mL Erlenmeyer flask and titrated with distilled water from a burette. The sample was swirled continuously at 150 rpm while being gradually titrated at a constant rate with water (0.05 mL/s) until the clear solution changed into a milky white fluid. The amount of water titrated was recorded and calculated into the percentage of water capacity using the equation (1) below (n = 3). This high value reflects the high water requirement to induce gel formation, meaning that the ISG solution exhibits a high water resistance or low sensitivity to water-induced gel formation.

(1) $$\%\mathrm{water}\;\mathrm{capacity}=\frac{amount\;of\;water\;titrated\;\left(mL\right)}{amount\;of\;water\;titrated\;\left(mL\right)+amount\;of\;ISG\;solution\;\left(mL\right)}\times 100$$

### 4.5. In Vitro Drug Release and Release Kinetics

A total of 0.4 g of ISG solution was added to a cylindrical-shaped porcelain cup (1 cm diameter and 1.2 cm height) that was then placed in the bottom of a tight glass bottle containing PBS pH 6.8 (80 mL). This bottle was constantly shaken at 50 rpm in a shaking incubator (model NB-205, N-Biotek, Gyeonggi-do, South Korea) at 37 °C to mimic the drug-release behavior from the crevicular pocket. The release medium was sampled (5 mL) for analysis and replaced with fresh PBS (5 mL). A UV–Visible spectrophotometer (Cary 60 UV–VIS, Model G6860A, Agilent, Selangor, Malaysia) was used to analyze the concentration of Dox in each sample (after filtering) at 280 nm. The percentage of cumulative drug release was calculated and plotted against time (n = 6). When the in vitro drug release process was complete (11 days), the porcelain cup containing the borneol gel was removed from the PBS, and the excess water in this cup was removed. Subsequently, the remnant remaining in the cup was observed under a stereo microscope (SZX10, Olympus Corp., Tokyo, Japan) with magnifications of 2.6× and 13.0× to study the morphology of the remnant gel after immersion in the release medium for 11 days.

The DD solver add-in program for Microsoft Excel (Redmond, WA, USA) was used for curve fitting to scrutinize the drug release kinetics from the obtained drug release profiles. Mathematical equations, including zero-order, first-order, Higuchi’s, Korsmeyer–Peppas’s, Hixon’s, Hofenberg’s, and Peppas–Sahlin’s equations, were used to fit the release profiles. The estimate-release parameters such as the coefficient of determination (R^2^), Akaike information criterion (AIC), and model selection criterion (MSC) were recorded. Typically, higher R^2^ and MSC, and lower AIC signify a better goodness-of-fit [67]. Furthermore, kinetic parameters were reported such as kKP (the constant of incorporation of structural modifications and geometrical characteristics of the system) and n (exponent of release) from the Korsmeyer–Peppas model [67]. In this model, n = 0.5 means that drug release is governed by diffusion or the Fickian model (Case I), whereas, n = 1.0 corresponds to zero-order release kinetics of the drug release rate, and the mechanism relates to the swelling or relaxation of the gel (Case II non-Fickian relaxational model). A value of n between 0.5 and 1.0 is applied to the non-Fickian model and the parameter from the Peppas–Sahlin’s model typically provides more detail regarding the drug release mechanism [67]. The parameters k_1_ (constant of Fickian diffusional contribution), k_2_ (constant of Case II relaxational contribution or swelling-erosion contribution), and m (the purely Fickian diffusion exponent) from Peppas–Sahlin’s model are practically employed to describe the drug release kinetics. The higher value for k_1_ or k_2_ indicates the mean of the dominant release mechanism of that formulation [67].

### 4.6. Antimicrobial Activities

The cylindrical cup agar plate technique was used to evaluate the antimicrobial activities of the ISG formulations. This evaluation technique is based on the diffusion of an active compound of the test formulation from a stainless steel cylinder cup (6 mm inside diameter and 10 mm height) through agar gel inoculated with the test microorganism. Microbes were calibrated for turbidity to a 0.5 McFarland standard before inoculation by spreading on TSA and SDA for bacteria and fungi, respectively. The sample from the ISG solution (100 μL) was added to a sterile cylinder cup, which was placed on the surface of an inoculated selected medium plate. Blood agar and chocolate agar were used for *P. gingivalis* ATCC 33,277 and *A. actinomycetemcomitans* ATCC 29522, respectively, and the experiments were performed in an anaerobic incubator (Forma Anaerobic System, Thermo Scientific, Ohio, USA). The inhibition zone as the clear diameter of each plate after incubation at 37 °C for 18 h was measured using a ruler (n = 3).

### 4.7. Statistical Analysis

One-way analysis of variance and Tukey’s test from the SPSS program (version 11.5) were performed to determine the statistical significance of the collected data. The significance level was chosen at *p* < 0.05.

## Figures and Tables

**Table 1:** Physical properties of ISG formulations (mean ± S.D.) (n = 3).

Formulation	Density (g/cm^3^)	Viscosity (cPs)	Surface Tension (mN/m)
Triacetin	1.1472 ± 0.0002	25.53 ± 0.52	38.87 ± 0.55
NMP	1.0283 ± 0.0004	1.88 ± 0.03	39.62 ± 0.02
N1	1.0425 ± 0.0009	2.59 ± 0.16	40.33 ± 0.42
N2	1.0204 ± 0.0014 ^a,b^	5.38 ± 0.09 ^e,f^	35.18 ± 0.16
N3	1.0228 ± 0.0011	5.55 ± 0.12	34.65 ± 0.23
N4	1.0268 ± 0.0017 ^a^	6.16 ± 0.07 ^e^	34.61 ± 0.22
N5	1.0290 ± 0.0009	6.66 ± 0.13	34.08 ± 0.17
N6	1.0340 ± 0.0014	7.06 ± 0.06	33.43 ± 0.07
N7	1.0386 ± 0.0017	8.84 ± 0.09	33.29 ± 0.08
N8	1.0435 ± 0.0013 ^b^	9.67 ± 0.04 ^f^	33.14 ± 0.09
DMSO	1.0924 ± 0.0003	2.25 ± 0.04	42.61 ± 0.46
D1	1.1090 ± 0.0003	3.07 ± 0.08	44.59 ± 0.36
D2	1.0524 ± 0.0009 ^c,d^	5.49 ± 0.12 ^g,h^	34.28 ± 0.95
D3	1.0538 ± 0.0008	5.92 ± 0.33	35.49 ± 0.15
D4	1.0557 ± 0.0013 ^c^	6.30 ± 0.19 ^g^	34.94 ± 0.38
D5	1.0579 ± 0.0009	7.19 ± 0.09	34.23 ± 0.38
D6	1.0601 ± 0.0004	8.19 ± 0.33	34.69 ± 0.15
D7	1.0626 ± 0.0011	8.76 ± 0.11	35.06 ± 0.25
D8	1.0656 ± 0.0006 ^d^	10.43 ± 0.62 ^h^	34.86 ± 0.31

The superscripts a–h indicate a significant difference (*p* < 0.05).

**Table 2:** Degree of goodness-of-fit and estimated parameters from the curve fittings to mathematical equations of Dox release profiles of different ISG formulations in PBS pH 6.8 (n = 3).

Formulation	Modeling	Criteria for Model Selection			Kinetic Parameters		
		*R* ^2^	AIC	MSC			
N1	Zero order	**0.9480**	32.8196	2.3843	k_0_ = 0.058		
	First order	**0.9831**	24.9566	3.5076	k_1_ = 0.001		
	Higuchi	0.9799	26.1750	3.3335	k_H_ = 2.578		
	Korsmeyer–Peppas	**0.9956**	17.5847	4.5607	k_KP_ = 9.011	n = 0.318	
	Hixson–Crowell	0.9833	33.9840	3.5898	k_HC_ = 0.0000		
	Hopfenberg	0.9921	30.0182	4.0855	k_HB_ = 0.0000	n = 185.258	
	Peppas–Sahlin	**0.9979**	21.2822	5.1775	**k_1_ = 8.630**	k_2_ = −0.048	m = 0.332
N2	Zero order	0.9111	90.8974	2.1756	k_0_ = 0.023		
	First order	**0.9889**	63.8739	4.2543	k_1_ = 0.000		
	Higuchi	0.9762	73.7490	3.4947	k_H_ = 1.253		
	Korsmeyer–Peppas	**0.9984**	40.7684	6.0316	k_KP_ = 0.461	n = 0.629	
	Hixson–Crowell	0.9812	70.7012	3.7291	k_HC_ = 0.000		
	Hopfenberg	0.9889	65.8890	4.0993	k_HB_ = 0.000	n = 653.884	
	Peppas–Sahlin	**0.9984**	42.7074	5.8825	k_1_ = −0.157	**k_2_ = 0.518**	m = 0.309
N4	Zero order	0.8408	121.9651	1.6132	k_0_ = 0.011		
	First order	0.9471	104.3304	2.7154	k_1_ = 0.000		
	Higuchi	**0.9969**	58.8696	5.5567	k_H_ = 0.889		
	Korsmeyer–Peppas	**0.9974**	57.9719	5.6128	k_KP_ = 0.761	n = 0.518	
	Hixson–Crowell	0.9288	109.0962	2.4175	k_HC_ = 0.000		
	Hopfenberg	0.9471	106.3399	2.5898	k_HB_ = 0.000	n = 1295.786	
	Peppas–Sahlin	**0.9987**	48.5514	6.2015	**k_1_ = 1.291**	k_2_ = 0.026	m = 0.399
N8	Zero order	0.9310	69.2509	2.3960	k_0_ = 0.036		
	First order	**0.9943**	41.8565	4.8864	k_1_ = 0.001		
	Higuchi	0.9411	67.5029	2.5550	k_H_ = 1.417		
	Korsmeyer–Peppas	**0.9985**	10.5336	5.7222	k_KP_ = 0.399	n = 0.679	
	Hixson–Crowell	0.9901	47.8600	4.3407	k_HC_ = 0.000		
	Hopfenberg	0.9943	43.7404	4.7152	k_HB_ = 0.000	n = 26.098	
	Peppas–Sahlin	**0.9986**	12.3015	5.3686	**k_1_ = 0.652**	k_2_ = 0.191	m = 0.376

**Table 3:** Inhibition zone diameter of different formulations against *P. gingivalis*, *A. actinomycetemcomitans*, MRSA, *S. aureus*, *E. coli*, *C. albicans*, *C. krusei*, *C. lusitaniae*, and *C. tropicalis* (n = 3).

Microbes	Inhibition Zone Diameter (Mean ± S.D.) (mm)					
	NMP	Triacetin	N1	N2	N4	N8
*P. gingivalis* ATCC 33277	15.00 ± 1.73 ^a,b^	NC	40.33 ± 1.53 ^a^	35.33 ± 0.58	34.33 ± 1.15 ^b^	32.67 ± 0.58
*A. actinomycetemcomitans* ATCC 29522	46.33 ± 0.58 ^c^	21.33 ± 0.58	50.00 ± 1.00 ^c^	37.33 ± 1.15	36.67 ± 0.58	30.67 ± 0.58
*S. aureus* (MRSA) ATCC 43300	17.00 ± 1.00 ^d,e^	12.33 ± 2.52	42.67 ± 1.15 ^d^	39.33 ± 1.15	38.00 ± 0.58 ^e^	36.67 ± 1.53
*S. aureus* ATCC 25923	18.67 ± 0.58 ^f,g^	NC	38.67 ± 0.58 ^f^	37.00 ± 1.00	35.33 ± 1.15 ^g^	33.33 ± 0.58
*E. coli* ATCC 8739	22.33 ± 0.58 ^h,i^	NC	29.33 ± 0.58 ^h^	28.67 ± 0.58	27.33 ± 0.00 ^i^	25.67 ± 0.58
*C. albicans* ATCC 17100	26.00 ± 1.00	12.00 ± 1.00	26.33 ± 0.58	22.33 ± 1.00	20.00 ± 0.00	17.33 ± 1.15
*C. krusei* TISTR 5259	31.30 ± 1.20	NC	32.33 ± 0.58	21.00 ± 1.00	18.33 ± 1.53	NC
*C. lusitaniae* TISTR 5156	38.33 ± 1.15	12.33 ± 0.58	38.33 ± 0.58	35.33 ± 1.15	34.33 ± 1.53	26.00 ± 1.00
*C. tropicalis* TISTR 5306	32.67 ± 0.58	NC	31.67 ± 0.58	29.67 ± 2.08	25.67 ± 1.53	18.00 ± 2.00

The superscripts ^a–i^ indicate a significant difference (*p* < 0.05); NC = the clear zone not found.

**Table 4:** Composition of Dox-loaded ISG formulations and control groups.

Formulation	Amount (% *w/w*)				
	Dox	Borneol	Triacetin	Solvent	
				NMP	DMSO
NMP	-	-	-	100	-
N1	5	-		95	-
N2	5	40	-	55	-
N3	5	40	2.5	52.5	-
N4	5	40	5.0	50	-
N5	5	40	10.0	45	-
N6	5	40	15.0	40	-
N7	5	40	20.0	35	-
N8	5	40	25.0	30	-
DMSO	-	-	-	-	100
D1	5	-	-	-	95
D2	5	40	-	-	55
D3	5	40	2.5	-	52.5
D4	5	40	5.0	-	50
D5	5	40	10.0	-	45
D6	5	40	15.0	-	40
D7	5	40	20.0	-	35
D8	5	40	25.0	-	30

## Data Availability

The datasets used and/or analyzed during the current study are available from the corresponding author upon reasonable request.
